# A Support Vector Machine Classification Model for Benzo[*c*]phenathridine Analogues with Topoisomerase-I Inhibitory Activity 

**DOI:** 10.3390/molecules17044560

**Published:** 2012-04-17

**Authors:** Khac-Minh Thai, Thuy-Quyen Nguyen, Trieu-Du Ngo, Thanh-Dao Tran, Thi-Ngoc-Phuong Huynh

**Affiliations:** Department of Medicinal Chemistry, School of Pharmacy, University of Medicine and Pharmacy at Ho Chi Minh City, 41 Dinh Tien Hoang St., District 1, Ho Chi Minh City, Vietnam; Email: tquyen172000@yahoo.com (T.-Q.N.); ngotrieudu1001@yahoo.com.vn (T.-D.N.); thanhdaot@yahoo.com (T.-D.T.); ngocphuonght@yahoo.com (T.-N.-P.H.)

**Keywords:** support vector machine, SVM, classification, topoisomerase, anticancer, benzo[c]phenanthridine, drug design, pharmacoinformatics

## Abstract

Benzo[*c*]phenanthridine (BCP) derivatives were identified as topoisomerase I (TOP-I) targeting agents with pronounced antitumor activity. In this study, a support vector machine model was performed on a series of 73 analogues to classify BCP derivatives according to TOP-I inhibitory activity. The best SVM model with total accuracy of 93% for training set was achieved using a set of 7 descriptors identified from a large set via a random forest algorithm. Overall accuracy of up to 87% and a Matthews coefficient correlation (MCC) of 0.71 were obtained after this SVM classifier was validated internally by a test set of 15 compounds. For two external test sets, 89% and 80% BCP compounds, respectively, were correctly predicted. The results indicated that our SVM model could be used as the filter for designing new BCP compounds with higher TOP-I inhibitory activity.

## 1. Introduction

**Figure 1:**
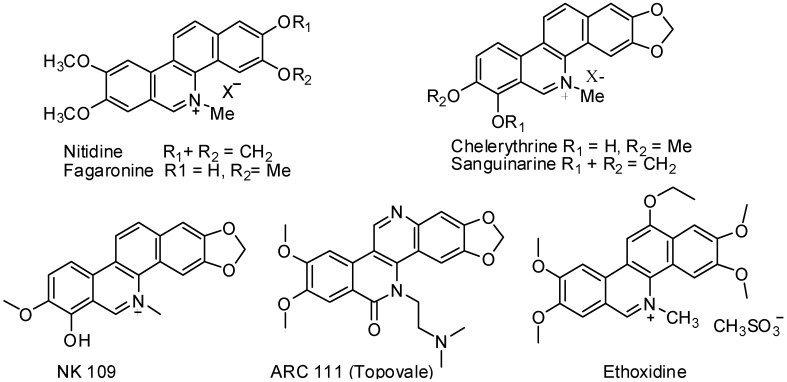
Chemical structures of benzo[*c*]phenanthridine derivatives.

The topoisomerases (TOP) are enzymes involved in processes such as replication, repair, transcription, recombination and segregation of DNA. Those of type I are the target of several anticancer agents based on their ability to stabilize the DNA-enzyme cleavage complex that causes DNA damage and cytotoxicity [1,2]. Among the agents expressing a targeted anti-topoisomerase activity, alkaloids of the benzo[*c*]phenanthridines (BCPs) family are well known [1,2,3,4]. Many BCP analogues were synthesized and evaluated for their activity on topoisomerase I as well as their cytotoxicity. Of those, ethoxidine, NK-109 and topovale (ARC 111) are potential candidates for cancer chemotherapy [1,2,3,4,5] (Figure 1).

The *in vitro* TOP-I inhibitory activity is valued by REC value, which is the relative effective concentration of TOP-I related to topotecan [6,7,8,9,10,11,12,13,14,15,16,17]. Therefore, compounds having TOP-I inhibitory activity could be divided into two classes of compounds based on the topotecan threshold. In this study, a support vector machine (SVM) approach was used to build up a classification model based on the anti-topoisomerase-I activity for BCP analogues. The SVM model could be applied to seek out new BCP analogues which are inhibitors of TOP-I for cancer treatment.

## 2. Results and Discussion

### 2.1. Feature Selection

Three feature selection approaches, namely mRMR (Max-Relevance, Min-Redundancy), GA (genetic algorithm) and RF (random forest) were applied to the dataset to figure out a set of chemical descriptors related to bioactivity properties from 2,032 molecular descriptors calculated by Dragon. Based on the calculation, three sets of molecular descriptors were selected, including a set of 10, 16 and seven descriptors for the mRMR, GA and RF methods, respectively. Table 1 shows the molecular descriptors selected via the RF method. After that, these sets of descriptors were used to develop the SVM models. For any SVM, it is necessary to select the optimal parameters of the Kernel function (C, γ) and these values for each set of descriptors were figured out and are shown in Table 2. The results of classification based on three different descriptor sets (by mRMR, GA and RF) by the SVM algorithm (the e1071 package in R) are shown in Table 3. The total accuracies of these SVM models are greater than or equal to 0.91 on training sets and are greater than or equal to 0.74 for cross-validation. However, the basic principle of SVM is a supervised learning approach. Hence, the classification results regarding test set and external set showed the relatively accurate performance of models. The SVM model which was developed from descriptors selected by RF method gave a better classification power than the two other methods. 

**Table 1:** Molecular descriptors from RF feature selection method.

Class	Symbol	Definition
Topological descriptors	D/Dr05	distance/detour ring index of order 5
	*D/Dr06*	distance/detour ring index of order 6
Walk and path counts	MPC06	molecular path count of order 06
	*MPC08*	molecular path count of order 08
	*MPC10*	molecular path count of order 10
2D frequency fingerprints	F05[C–C]	frequency of C–C at topological distance 05
	*F08[N*–*O]*	frequency of N–O at topological distance 08

**Table 2:** Optimal parameters (C, γ) for SVM approach.

Feature selection method	Number of features selected	Range of γ	Best γ	Range of C	Best C	Cross-validation error
mRMR	10	5^[−10:10]^	3.125	5^[−10:10]^	1	0.21
GA	16	2^[−10:10]^	0.125	2^[−10:10]^	16	0.15
RF	7	2^[−10:10]^	0.25	2^[−10:10]^	4	0.17

**Table 3:** Classification results of 3 SVM models corresponding with 3 descriptor sets selected successively via mRMR, GA and RF methods.

Feature selection method	Training set			Test set			External set		
	mRMR	GA	RF	mRMR	GA	RF	mRMR	GA	RF
Number of support vectors	51	40	35						
Total accuracy	0.98	0.91	0.93	0.80	0.93	0.87	0.67	0.78	0.89
Sensitivity	0.93	0.73	0.87	0.33	1.00	1.00	0.40	0.60	0.80
Specificity	1.00	0.98	0.95	0.92	0.92	0.83	1.00	1.00	1.00
Positive precision	1.00	0.92	0.87	0.50	0.75	0.60	1.00	1.00	1.00
Negative precision	0.98	0.91	0.95	0.85	1.00	1.00	0.57	0.67	0.80
Matthews correlation cofficient (MCC)	0.96	0.77	0.82	0.29	0.83	0.71	0.48	0.63	0.80
Total accuracy of cross-validation	0.76	0.78	0.74						

### 2.2. SVM Classification Model

A total of 82 compounds were used for SVM and classified into actives or inactives based on the the relative effective concentration (REC) compared to topotecan and the respective threshold points. For the separation of active/inactive, REC of topotecan (REC = 1) was selected as a threshold point. TOP-I active compound having equal or stronger activity than that of topotecan is presented as “1” and *vice versa*, “0” is presented TOP-I inactive compound having weaker activity than that of topotecan. The set of molecular descriptors selected via RF method was used to create the final SVM (the e1071 package in R) classification model based on anti-topoisomerase-1 activity. Moreover, two other classification approaches namely the SVM kernlab and “randomForest” (RF) in R are also applied and the classification results are presented in Table 4. In general, both of SVM packages (the e1071 package, the package kernlab) gave better results than those of observed for random forest methods with the different values in the total accuracy of 20%. 

**Table 4:** Classification results from three classified approaches.

	Training set			Test set			External set			Application set
Evaluation criteria	SVM e1071	SVM Kernlab	RF	SVM e1071	SVM kernlab	RF	SVM e1071	SVM kernlab	RF	SVM e1071
TP	**13**	13	6	**3**	3	3	**4**	3	3	**5**
TN	**41**	41	36	**10**	8	10	**4**	4	3	**3**
FP	**2**	2	9	**0**	0	0	**1**	2	2	**0**
FN	**2**	2	7	**2**	4	2	**0**	0	1	**2**
Total accuracy	**0.93**	0.93	0.72	**0.87**	0.73	0.87	**0.89**	0.78	0.67	**0.80**
Sensitivity	**0.87**	0.87	0.46	**0.60**	0.43	0.60	**1.00**	1.00	0.75	**0.71**
Specificity	**0.95**	0.95	0.80	**1.00**	1.00	1.00	**0.80**	0.67	0.60	**1.00**
Positive precision	**0.87**	0.87	0.40	**1.00**	1.00	1.00	**0.80**	0.60	0.60	**1.00**
Negative precision	**0.95**	0.95	0.84	**0.83**	0.67	0.83	**1.00**	1.00	0.75	**0.60**
MCC	**0.82**	0.82	0.25	**0.71**	0.54	0.71	**0.80**	0.63	0.35	**0.65**
Cross-validation error	**0.22**	0.20	0.28 ^a^							
Y-scrambling total accuracy	**0.59**									

^a^ “out of bags” of RF method is based on principle of cross-validation.

Regarding the training set, two SVM packages (e1071, kernlab) expressed similar classification power. However, regarding the test set and the external set, the SVM package e1071 showed the better results. The Matthews correlation coefficient (MCC) of this package was stable for training, test and external test sets with values 0.82, 0.71 and 0.80, respectively. For the kernlab package, the MCC value for the training set was as high as 0.82 whereas the MCC of the test set was only 0.54. Normally, a model with MCC larger than 0.4 indicates that it has the predictive power [18]. The RF classification method in combination with a set of descriptors chosen by RF gave MCC = 0.35 on the external set and this result indicates that the RF classification model has no ability to predict the biological activity of compounds in this study. The SVM-e1071 in R classification model with a set of descriptors identified from a large set via a RF algorithm showed the best results.

To further validate the use of SVM-e1071 for TOP-I classification, a 50-fold Y-scrambling procedure for the training sets was performed [19,20,21]. The total accuracy of 0.59 was obtained from the Y-scrambling analyses. The results showed that our models show a significantly better performance than those obtained when class assignments are randomly achieved with total accuracy values of 0.93 versus 0.59, respectively. However, it has to be noted that the principle of SVM is supervised learning and the learning strategy tries to keep the error to a minimum values which explains the relative “good” performance of this method for the y scrambled data sets.

### 2.3. Validation and Application

The final SVM model was validated not only by cross-validation procedure but also by an external dataset (not belonging to the dataset used to create the SVM classification model). The power for classification of BCPs by anti-TOP-I activity was 0.87–0.93 for total accuracy and 0.22 for cross-validation error. Moreover, the final SVM model was applied to classify a set of 10 BCP analogues recently synthesized by Lavoie *et al.*, which so-called application set or external test set 2 and detailed chemical structures and TOP-I activities are shown in Table 5 [22,23,24,25,26]. According to literature, among these 10 BCP analogues, seven compounds have stronger anti-TOP-I activity than topotecan and three compounds are weaker than topotecan. The results indicated that the classification model was achieved a correct prediction of 80% (8/10) and the detailed results are presented in Table 4. The positive accuracy gained the value of 100% *i.e*., the classification model is more accurate for predicting compounds wtith stronger activity than topotecan. 

**Table 5:** Chemical structure often benzo[*c*]phenanthridine derivatives in application set and their topoisomerase I inhibitory activity REC and classification results from final SVM model. Classification term: “1” presented TOP-I active compound having equal or stronger activity than that of topotecan; “0” presented TOP-I inactive compound having weaker activity than that of topotecan.

No	Chemical structure	Name	REC TOP-I mediated DNA cleavage (Experimental)	TOP-I Active/Inactive	TOP-I classified result from final SVM model
A1	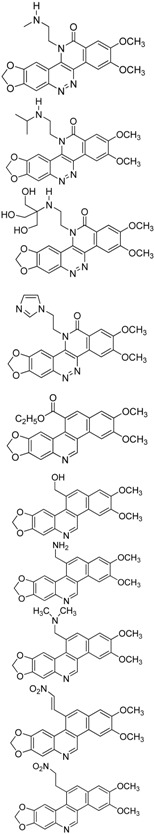	BMC_08_7824_7a	0.03	1	1
A2		BMC_08_7824_7b	0.08	1	1
A3		BMC_08_7824_9	0.2	1	1
A4		BMC_08_7824_11	0.1	1	1
A5		BMC_08_8598_9	>10	0	0
A6		BMC_08_8598_10	0.2	1	0
A7		BMC_08_8598_12	0.2	1	0
A8		BMC_08_8598_13	0.2	1	1
A9		BMC_08_8598_14	>10	0	0
A10		BMC_08_8598_15	>10	0	0

This result completely meets our goal, which is aiming to look for new inhibitors of TOP-I for cancer chemotherapy. However, one limitation of all machine learning approaches is their inability to indicate the important role of functional groups related to biological activity. Hence, the combination of this SVM classification model with molecular docking studies [27,28] and also related 2D- and 3D-QSAR model on cytotoxicity of BCPs [5] could provide insight into the molecular basis of TOP-I inhibitors.

### 2.4. Discussions

In this study, the TOP-I inhibitory activity of topotecan, the synthetic derivative of camptothecin and the most potent anticancer drugs in clinical use, is used as threshold points for SVM classification models. Topotecan, ethoxidine, fagaronine and BCP related compounds indicated the selectivity on TOP-I than TOP-II. These novels acted as DNA intercalators and having two mechanisms including *(i)* TOP-I poison as fagaronine; and *(ii)* TOP-I suppressor as ethoxidine [27,28]. Our preliminary results from *in silico* modeling indicated that BCP compounds may inhibit the TOP-I activity via suppression mechanism.

SVM is a machine learning method, which has been used for many kinds of pattern recognition problems. SVMs have been successfully adapted to treat both regression and classification [29]. In this study, mRMR, GA and RF algorithms were used for the selection of descriptors from large descriptors set derived from Dragon software and the results showed that the descriptors selected from the RF method gave a better classification power than others. The SVM also indicated the better results on classification of BCP analogues with anti-TOP-I activity than that of RF. This final SVM model with its high accuracy (80–90%), fast calculation without the need of 3D conformation and accurate prediction on the substances having positive activity could be applied to look for and design new analogues of BCPs with higher topoisomerase I inhibitory activity. SVMs are a black box technique and deliver information about their predictions other than the relationship between molecular descriptors and bioactivity. The SVM model’s explanatory in term molecular descriptors and bioactivity could be performed if the weights of each descriptor are explicitly solved [29]. It should be noted that our SVM model could be applied in screening prior to synthesis procedures for new BCP-like compouds to identify potential TOP-I inhibitors. However, the development of a useful drug has to be dealt on systems level of drug discovery and development and take into consideration many factors including solubility, ease of drug formulation, selectivity and ADME-Tox. 

## 3. Experimental

### 3.1. Dataset

BCP analogues in the study (82 compounds) with topoisomerase I inhibitory activity evaluated by testing of DNA cleavage were collected from the work of LaVoie *et al.* [6,7,8,9,10,11,12,13,14,15,16,17]. The biological activity data is represented by the relative effective concentration (REC) compared to topotecan whose value is arbitrarily set at 1.0 (as reference compound). This parameter allows the comparison of molecules based on the cleavage of plasmid DNA in the presence of human topoisomerase I. Therefore, with a compound having the REC value higher than 1, that means its topoisomerase I inhibitory activity lower than topotecan and vice versa. Based upon the REC values, the data set was grouped into two classes: 58 analogues were assigned to inactive class (REC value > 1, negatives or inactives) and 24 analogues were classified as active class with REC value ≤ 1 (high TOP-I blockade, positives or actives). Chemical structure of 82 benzo[*c*]phenanthridine derivatives and their topoisomerase I inhibitory activity REC values were presented in Table 6. 

**Table 6:** Chemical structure of82 benzo[*c*]phenanthridine derivatives and their topoisomerase I inhibitory activity REC and classification results from final SVM model. Classification term: “1” presented TOP-I active compound having equal or stronger activity than that of topotecan; “0” presented TOP-I inactive compound having weaker activity than that of topotecan.

No	Chemical structure	Name	REC TOP-I mediated DNA cleavage (Experimental)	TOP-I Active/Inactive	TOP-I classified result from final SVM model
1	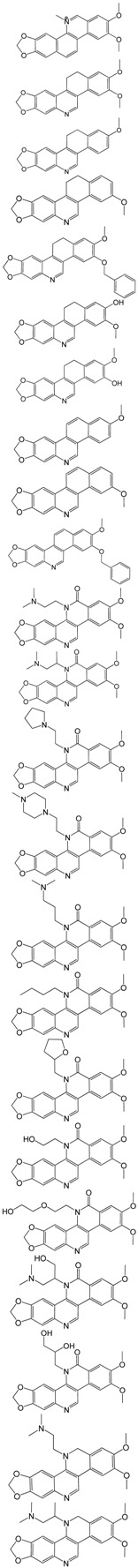	Nitidine	10	0	0
2		BMC_03_3795_10a	8	0	0
3		BMC_03_3795_10b	200	0	0
4		BMC_03_3795_10c	200	0	0
5		BMC_03_3795_10d	>1000	0	0
6		BMC_03_3795_10e	500	0	0
7		BMC_03_3795_10f	10	0	0
8		BMC_03_3795_11a	>1000	0	0
9		BMC_03_3795_11b	100	0	0
10		BMC_03_3795_12d	>1000	0	0
11		BMC_03_2061_03a	0.5	1	0
12		BMC_03_2061_03b	>1000	0	0
13		BMC_03_2061_03c	0.3	1	1
14		BMC_03_2061_03d	1000	0	0
15		BMC_03_2061_03e	1	1	0
16		BMC_03_2061_03f	1000	0	0
17		BMCL_02_3333_03c	10	0	0
18		BMC_03_2061_03h	50	0	0
19		BMC_03_2061_03i	1	1	0
20		BMC_03_2061_03j	>1000	0	0
21		BMC_03_2061_03k	0.8–1.0	1	1
22		BMC_03_2061_04a	0.8	1	0
23		BMC_03_2061_04b	100	0	1
24	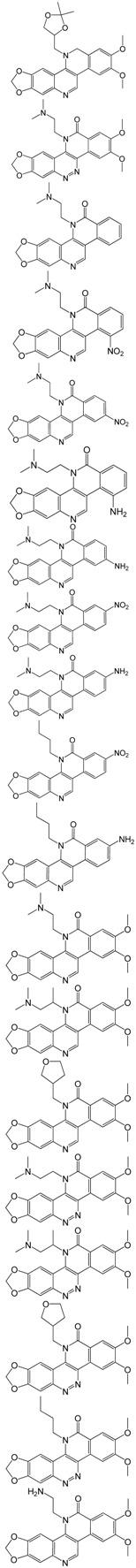	BMC_03_2061_09k	10	0	0
25		JMC_03_2254_02	0.3	1	0
26		JMC_03_2254_03	6	0	0
27		JMC_03_2254_05a	1000	0	1
28		JMC_03_2254_05b	0.1	1	1
29		JMC_03_2254_06a	15	0	0
30		JMC_03_2254_06b	0.5	1	0
31		JMC_03_2254_05c	0.2	1	0
32		JMC_03_2254_06c	8.0	0	0
33		JMC_03_2254_16a	10	0	1
34		JMC_03_2254_17a	500	0	0
35		JMC_03_2254_02	0.5	1	1
36		BMCL_02_3333_02	>1000	0	0
37		BMCL_02_3333_03	200	0	0
38		BMCL_02_3333_04a	0.3	1	1
39		BMCL_02_3333_04b	1000	0	0
40		BMCL_02_3333_04c	30	0	0
41		BMCL_02_3333_04d	1.0	1	0
42		LDDD_04_198_01	0.03	1	0
43	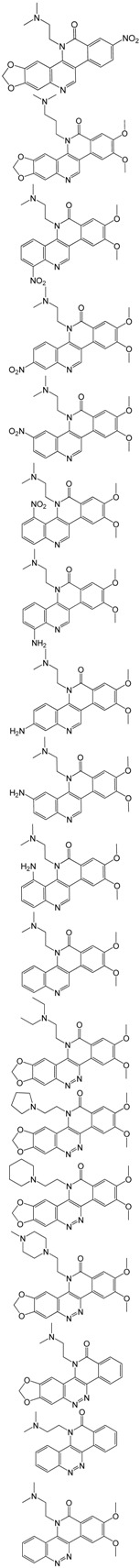	LDDD_04_198_02	2.0	0	0
44		LDDD_04_198_03	2.0	0	0
45		BMC_04_3731_03a	9	0	0
46		BMC_04_3731_03b	6	0	0
47		BMC_04_3731_03c	2	0	0
48		BMC_04_3731_03d	>300	0	0
49		BMC_04_3731_04a	100	0	0
50		BMC_04_3731_04b	12	0	0
51		BMC_04_3731_04c	6	0	0
52		BMC_04_0795_04	>300	0	0
53		BMC_04_3731_05	10	0	0
54		BMC_04_0795_01b	0.3	1	0
55		BMC_04_0795_01f	0.2	1	1
56		BMC_04_0795_01g	2.0	0	0
57		BMC_04_0795_01h	20	0	0
58		BMC_04_0795_03	10	0	0
59		BMC_04_0795_02	>1000	0	0
60		BMC_04_0795_04	0.8	1	1
61	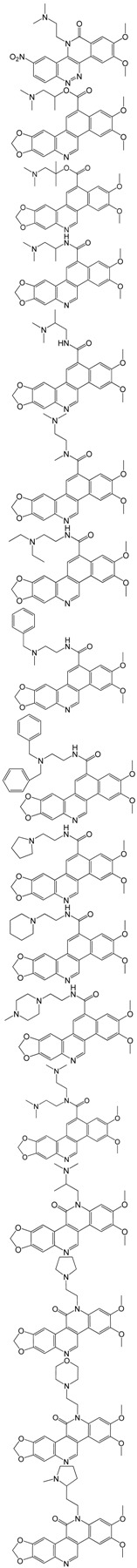	BMC_04_0795_05	5	0	0
62		BMC_05_6782_07c	1.0	1	0
63		BMC_05_6782_07d	8	0	0
64		BMC_05_6782_08	0.6	1	1
65		BMC_05_6782_09c	0.4	1	0
66		BMC_05_6782_09d	10	0	0
67		BMC_05_6782_09e	0.7	1	1
68		BMC_05_6782_09f	12	0	0
69		BMC_05_6782_09g	60	0	0
70		BMC_05_6782_09h	0.6	1	1
71		BMC_05_6782_09i	1.5	0	0
72		BMC_05_6782_09j	0.3	1	1
73		BMC_05_6782_10	3	0	1
74		BMC_06_3131_10c	1.2	0	0
75		BMC_06_3131_10d	0.07	1	1
76		BMC_06_3131_10g	9	0	0
77		BMC_06_3131_10i	0.45	1	1
78	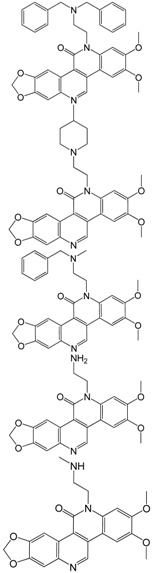	BMC_06_3131_10j	>100	0	0
79		BMC_06_3131_10f	3	0	0
80		BMC_06_3131_10k	13	0	0
81		BMC_06_3131_10l	0.35	1	1
82		BMC_06_3131_10m	0.15	1	1

### 3.2. Training and Test Sets

The training and test sets were generated by random division. Firstly, a set of nine compounds was selected and not used to develop the models. These compounds were separated from the others and considered as external set. The remaining analogues were split randomly for five times into 80% for training sets and 20% for test sets using the R software [30,31]. The numbers of compounds in each subset are presented in Table 7.

**Table 7:** BCPs datasets division.

Dataset	Total compounds	Actives ^a^	Inactives ^b^
Whole set	82	24	58
Training set	58	15	43
Test set	15	5	10
External set	9	4	5
Application set (External set 2)	10	7	3

^a^ Actives: Compounds whose activity is equal or stronger than topotecan; ^b^ Inactives: compounds whose activity is weaker than topotecan.

### 3.3. Molecular Descriptors and Feature Selection

The Dragon software was applied to calculate 2,032 molecular descriptors [32,33]. In this study, several feature selection algorithms were performed to reduce dimensionality of descriptor space. Firstly, chemical descriptors only containing the value of zero were eliminated before using the mathematic methods. Only 533 descriptors were selected for next steps. In addition, highly correlated descriptors (r > 0.90) were removed to avoid redundancy and to manage the data more efficiently in terms of computation resources and intuitive perception of the chemical space. A total of 103 descriptors were selected and scaled to unit variance to be used as input value to SVMs. Finally, three feature selection methods namely mRMR (Max-Relevance, Min-Redundancy), GA (genetic algorithm) and RF (random forest) were applied in order to select the optimum set for the molecular descriptors [30,34].

### 3.4. Support Vector Machine

**Figure 2:**
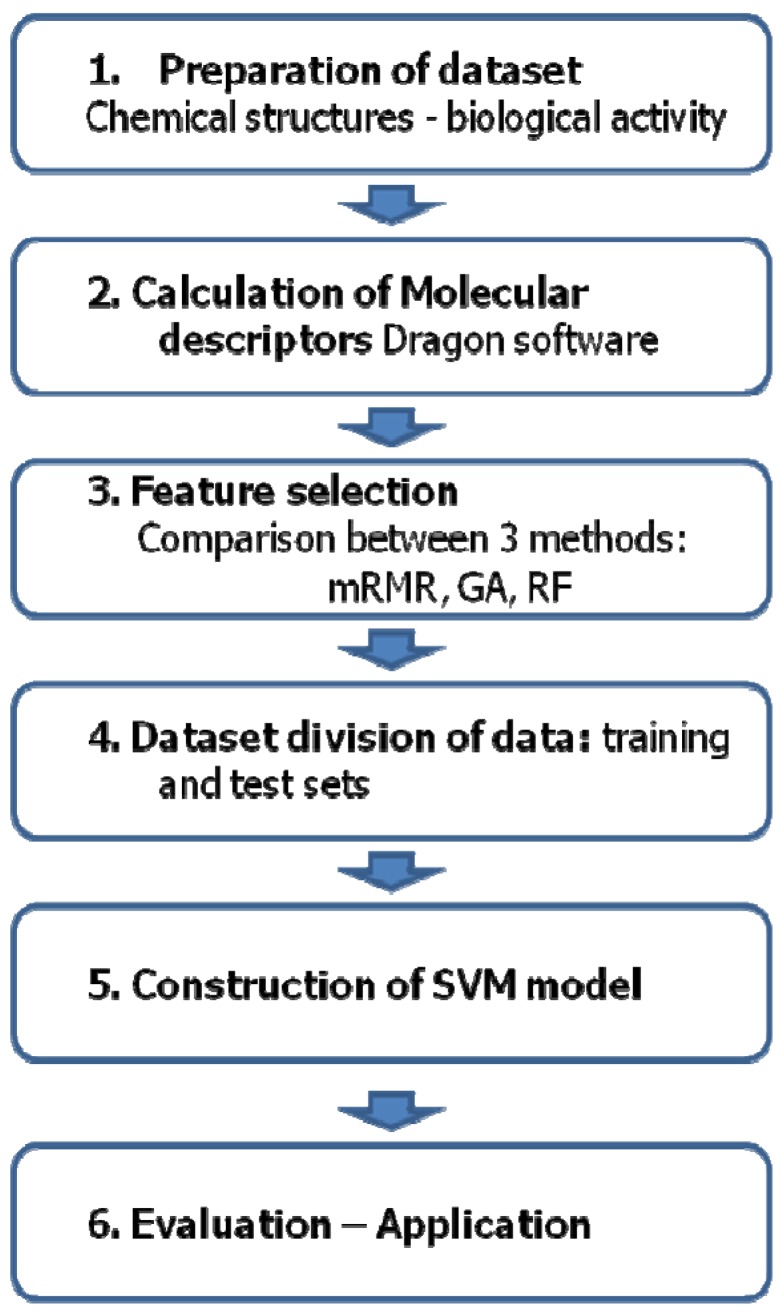
Process of SVM classification model.

Support vector machine (SVM), the most successfully applied new classification algorithms, were introduced to the machine learning community by Vapnik. Smola and Schölkopf provided an extensive tutorial on SVMs [30,31,32,33,34]. The underlying idea of an SVM classifier is to map linearly inseparable input data into a higher dimensional space where the data can be linearly separated, using a maximal separating hyperplane. Support Vector Machines (SVM) is the classification system based on the supervised learning approach. In this study, SVM algorithm in the e1071 package in R with Kernel function was used [30,31]. The strategies for the SVM classification model are shown in Figure 2.

**Figure 3:**
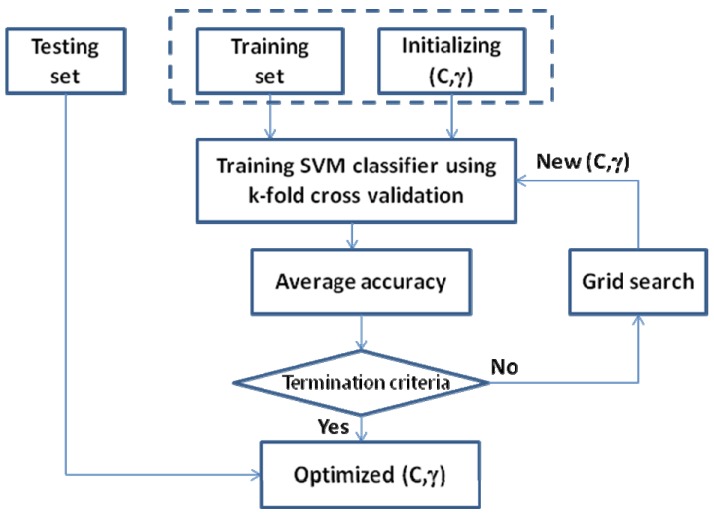
Selection of optimal parameters of Kernel function using Grid algorithm.

Selection of optimal parameters of Kernel function: RBF Kernel function has two parameters (C and γ) and the selection of these parameters is one of two critical issues to develop the good SVM model (the other is the feature selection) [35,36,37]. To find the optimal parameters, an algorithm Grid (function tune in R) is made following the process described in Figure 3. The training set and test set are used to find a pair of optimal parameters (C and γ) of the Kernel function. Pairs of parameters were tested in intervals reduced step by step an algorithm Grid. The pair is chosen when the error of cross validation is minimal [30,34].

### 3.5. Evaluation Criteria for Classification Model

Performance of the SVM models was measured by using standard parameters for classification models [18,19,38,39,40]. They are described as follows:

(i) The overall classification accuracy of a prediction model, accuracy = (tp + tn)/(tp + fp + tn + fn),
(ii) Sensitivity (recall, accuracy on actives) = tp/(tp + fn),
(iii) Specificity (accuracy on inactives) = tn/(tn + fp),
(iv) Precision on actives = tp/(tp + fp),
(v) Precision on inactives = tn/(tn + fn) and,
(vi) Matthews correlation coefficient (MCC):

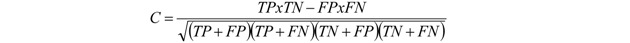


The quality of a classification model is generally measured by using these parameters, which are estimated for the whole set and by applying a cross-validation protocol based on a leave-one-out (LOO) procedure. In all equations, tp = number of true positives, tn = number of true negatives, fp = number of false positives, and fn = number of false negatives.

## 4. Conclusions

In this study, the SVM was used to build up a model for prediction and classification of 73 BCP analogues based on their anti-topoisomerase-1 activity. The best model was derived from the SVM-e1071 package in R with the optimal settings of the Kernel function (C = 4, γ = 0.25) and the set of descriptors selected by RF method. This final SVM model is able to correctly predict the anti-topoisomerase activity for 93% of compounds in the training set and 87% of those in a test set. It was also validated on external sets not involving in the dataset used with the developed model and then on the application set. Total accuracies of 89% (the prediction is correct for eight out of nine compounds) and 80% (the prediction is correct for eight out of 10 compounds) were obtained for the external set and application sets, respectively. Furthermore, this model has also proved its ability to classify correctly BCP analogues that have the positive activity, with an accuracy from 80 to 100% overall. With its high accuracy (80–90%), fast and accurate prediction on the substances having positive activity, our SVM model could be applied to look for and design new analogues of BCPs with higher topoisomerase I inhibitory activity. 

## Supplementary Materials

Supplementary materials can be accessed at: http://www.mdpi.com/1420-3049/17/4/4560/s1.

## References

[1] Fleury F., Sukhanova A., Ianoul A., Devy J., Kudelina I., Duval O., Alix A.J.P., Jardillier J.C., Nabiev I. (2000). Molecular determinants of site-specific inhibition of human DNA topoisomerase I by fagaronine and ethoxidine. Relation to DNA binding. J. Biol. Chem..

[2] Pommier Y. (2009). DNA topoisomerase I inhibitors: Chemistry, biology, and interfacial inhibition. Chem. Rev..

[3] Nakanishi T., Suzuki M., Mashiba A., Ishikawa K., Yokotsuka T. (1998). Synthesis of NK109, an anticancer benzo[*c*]phenanthridine alkaloid. J. Org. Chem..

[4] Li T.K., Houghton P.J., Desai S.D., Daroui P., Liu A.A., Hars E.S., Ruchelman A.L., LaVoie E.J., Liu L.F. (2003). Characterization of ARC-111 as a novel topoisomerase I-targeting anticancer drug. Cancer Res..

[5] Liao S.Y., Qian L., Lu H.L., Shen Y., Zheng K.C. (2008). A combined 2D- and 3D-QSAR study on analogues of ARC-111 with antitumor activity. QSAR Comb. Sci..

[6] Li D., Zhao B., Sim S.-P., Li T.-K., Liu A., Liu L.F., LaVoie E.J. (2003). 2,3-Dimethoxybenzo(i)-phenanthidines: Topoisomerase I-targeting anticancer agents. Bioorg. Med. Chem..

[7] Yu Y., Singh S.K., Liu A., Li T.K., Liu L.F., LaVoie E.J. (2003). Substituted dibenzo(*c*,*h*)cinnolines: Topoisomerase I-targeting anticancer agents. Bioorg. Med. Chem..

[8] Makhey D., Li D., Zhao B., Sim S.-P., Li T.-K., Liu A., Liu L.F., LaVoie E.J. (2003). Substituted benzo(*i*)phenanthridines as mammalian topoisomerase I-targeting anticancer agents. Bioorg. Med. Chem..

[9] Ruchelman A.L., Singh S.K., Ray A., Wu X.H., Yang J.-M., Li T.-K., Liu A., Liu L.F., LaVoie E.J. (2003). 5H-Dibenzo(c,h)1,6-napthiridin-6-ones: Novel topo-isomerase I-targeting anticancer agents with potent cytotoxic activity. Bioorg. Med. Chem..

[10] Li D., Zhao B., Sim S.-P., Li T.-K., Liu A. (2003). 8,9-Methylenedioxybenzo-(*i*)phenanthridines: Topoisomerase I-targeting activity and cytotoxicity. Bioorg. Med. Chem..

[11] Ruchelman A.L., Singh S.K., Ray A., Wu X., Yang J.M., Zhou N., Liu A., Liu L.F., LaVoie E.J. (2004). 11*H*-Isoquino(4,3-*c*)cinnolin-12-ones: Novel anticancer agents with potent topoisomerase I-targeting activity and cytotoxicity. Bioorg. Med. Chem..

[12] Ruchelman A.L., Kerrigan J.E., Li T.-K., Zhou N., Liu A., Liu L.F., LaVoie E.J. (2004). Nitro and amino substitution within the A-ring of 5*H*-8,9-dimethoxy-5-(2-*N*,*N*-dimethylamino-ethyl)dibenzo(*c*,*h*)(1,6)naph-thyridin-6-ones: Influence on topoisomerase I-targeting activity and cytotoxicity. Bioorg. Med. Chem..

[13] Zhu S., Ruchelman A.L., Zhou N., Liu A., Liu L.F., LaVoie E.J. (2005). Esters and amides of 2,3-dime-thoxy-8,9-methylene-dioxybenzo(*i*)phenanthridine-12-carboxylic acid: Potent topoisomerase I-targeting agents. Bioorg. Med. Chem..

[14] Zhu S., Ruchelman A.L., Zhou N., Liu A., Liu L.F., LaVoie E.J. (2006). 6-Substituted 6*H*-dibenzo(*c*,*h*)(2,6)naph-thyridin-5-ones: Reversed lactam analogues of ARC-111 with potent topoisomerase I-targeting activity and cytotoxicity. Bioorg. Med. Chem..

[15] Ruchelman A.L., Singh S.K., Wu X., Ray A., Yang J.M., Li T.-K., Liu A., Liu L.F., LaVoie E.J. (2002). Diaza- and triazachrysenes: Potent topoisomerase I-targeting agents with exceptional antitumor activity against the human tumor xenograf, MDA-MB-435. Bioorg. Med. Chem. Lett..

[16] Ruchelman A.L., Zhu S., Zhou N., Liu A., Liu L.F., LaVoie E.J. (2004). Dimethoxybenzo(*i*)-phenanthridine-12-carboxylic acid derivatives and 6*H*-dibenzo(*c*,*h*) (2,6)naphthyridin-5-ones with potent topoisomerase I-targeting activity and cytotoxicity. Bioorg. Med. Chem. Lett..

[17] Feng W., Satyanarayana M., Cheng L., Liu A., Tsai Y.-C., Liu L.F., LaVoie E.J. (2008). Synthesis of N-substituted 5-[2-(*N*-alkylamino)ethyl]dibenzo[*c*,*h*][1,6]-naphthyridines as novel topoisomerase I-targeting anti-tumor agents. Bioorg. Med. Chem..

[18] Yap C.W., Chen Y.Z. (2005). Prediction of cytochrome P450 3A4, 2D6, and 2C9 Inhibitors and substrates by using support vector machines. J. Chem. Inf. Model..

[19] Thai K.-M., Ecker G.F. (2008). Classification models for hERG inhibitors by counter-propagation neural networks. Chem. Biol. Drug Des..

[20] Kubinyi H., Sener E.A., Yalcin I. (2006). Validation and predictivity of QSAR models. QSAR & Molecular Modelling in Rational Design of Bioactive Molecules, Proceedings of the 15th European Symposium on QSAR & Molecular Modelling, Istanbul, Turkey, 2004.

[21] Gramatica P. (2007). Principles of QSAR models validation: Internal and external. QSAR Comb. Sci..

[22] Feng W., Satyanarayana M., Tsai Y.-C., Liu A.A., Liu L.F., LaVoie E.J. (2008). 11-Substituted 2,3-dimethoxy-8,9-methylenedioxybenzo[*i*]phenanthridine derivatives as novel topoisomerase I-targeting agents. Bioorg. Med. Chem..

[23] Satyanarayana M., Tsai Y.-C., Liu A.A., Liu L.F., LaVoie E.J. (2008). Syntheses and biological evaluation of topoisomerase I-targeting agents related to 11-[2-(*N*,*N*-dimethylamino)ethyl]-2,3-dimethoxy-8,9-methylene-dioxy-11*H*-isoquino[4,3-*c*]cinnolin-12-one (ARC-31). Bioorg. Med. Chem..

[24] Satyanarayana M., Tsai Y.-C., Liu A.A., Liu L.F., LaVoie E.J. (2008). Synthesis of N-substituted 5-[2-(N-alkyl-amino)ethyl]-dibenzo[*c*,*h*][1,6]-naphthyridines as novel topoisomerase I-targeting antitumor agents. Bioorg. Med. Chem..

[25] Scaglioni L., Mazzini S., Mondelli R., Dallavalle S., Gattinoni S., Tinelli S., Beretta G.L., Zunino F., Ragg E. (2009). Interaction between double helix DNA fragments and a new topopyrone acting as human topoisomerase I poison. Bioorg. Med. Chem..

[26] Satyanarayana M., Tsai Y.-C., Liu A.A., Liu L.F., LaVoie E.J. (2009). 12-Substituted 2,3-dimethoxy-8,9-methylenedioxybenzo-[*i*]phenanthridines as novel topoisomerase I-targeting antitumor agents. Bioorg. Med. Chem..

[27] Clark R.L., Deane F.M., Anthony N.G., Johnston B.F., McCarthy F.O., Mackay S.P. (2007). Exploring DNA topoisomerase I inhibition by the benzo[*c*]phenanthridines fagaronine and ethoxidine using steered molecular dynamics. Bioorg. Med. Chem..

[28] Khadka D.B., Cho W.J. (2011). 3-Arylisoquinolines as novel topoisomerase I inhibitors. Bioorg. Med. Chem..

[29] Muegge I., Oloff S. (2006). Advances in virtual screening. Drug Dis. Today Tech..

[30] The R Project for Statistical Computing. http://www.r-project.org.

[31] Karatzoglou A., Meyer D., Hornik K. (2006). Support vector machines in R. J. Stat. Softw..

[32] Tetko I.V., Gasteiger J., Todeschini R., Mauri A., Livingstone D., Ertl P., Palyulin V.A., Radchenko E.V., Zefirov N.S., Makarenko A.S. (2005). Virtual computational chemistry laboratory—Design and description. J. Comput. Aid. Mol. Des..

[33] VCCLAB, Virtual Computational Chemistry Laboratory. http://www.vcclab.org.

[34] Hsu C.-W., Chang C.-C., Lin C.-J. LIBSVM: A Practical Guide to Support Vector Classification. http://www.csie.ntu.edu.tw/~cjlin/libsvm.

[35] Fröhlich H., Chapelle O., Schölkopf B. (2003). Feature selection for support vector machines by means of genetic algorithms. In. Proceeding ICTAI '03, Proceedings of the 15th IEEE International Conference on Tools with Artificial Intelligence.

[36] Fröhlich H., Chapelle O., Schölkopf B. (2004). Feature selection for support vector machines using genetic algorithms. Int. J. Artif. Intell. T..

[37] Demel M.A., Janecek A.G.K., Thai K.-M., Ecker G.F., Gansterer W.N. (2008). Predictive QSAR models for polyspecific drug targets: The importance of feature selection. Curr. Comput. Aided Drug Des..

[38] Thai K.-M., Ecker G.F. (2008). A binary QSAR model for classification of hERG potassium channel blockers. Bioorg. Med. Chem..

[39] Thai K.-M., Ecker G.F. (2009). Similarity-based SIBAR descriptors for classification of chemically diverse hERG blockers. Mol. Divers..

[40] Ji L., Wang X., Qin L., Luo S., Wang L. (2009). Predicting the androgenicity of structurally diverse compounds from molecular structure using different classifiers. QSAR Comb. Sci..

