# Supplementary material for: A Support Vector Machine Classification Model for Benzo[c]phenathridine Analogues with Topoisomerase-I Inhibitory Activity
Source: Molecules. 2012 Apr 17;17(4):4560–82. doi: 10.3390/molecules17044560 (PMC6268465; doi:10.3390/molecules17044560)
Supplement: Supplementary file 1 [file molecules-17-04560-s001.pdf]

Supplementary Material

Classification Model for Benzo[*c*]phenathridine Analogues with Toposiomerase-I Inhibitory Activity by Support Vector Machine

Khac-Minh Thai *, Thuy-Quyen Nguyen, Trieu-Du Ngo, Thanh-Dao Tran and Thi-Ngoc-Phuong Huynh

Department of Medicinal Chemistry, School of Pharmacy, University of Medicine and Pharmacy at Ho Chi Minh City, 41 Dinh Tien Hoang St., District 1, Ho Chi Minh City, Viet Nam; E-Mails: tquyen172000@yahoo.com (T.-Q.N.); ngotrieudu1001@yahoo.com.vn (T.-D.N.); thanhdaot@yahoo.com (T.-D.T.); ngocphuonght@yahoo.com (T.-N.-P.H.)

* Author to whom correspondence should be addressed; E-Mails: thaikhacminh@gmail.com or thaikhacminh@uphcm.edu.vn; Tel.: +84-909-680-385; Fax: +84-838-225-435.

Table S1. Chemical structure of 82 benzo[*c*]phenanthridine derivatives and their topoisomerase I inhibitory activity REC and classification results from the final SVM model.

Table S2. Chemical structure of 10 benzo[*c*]phenanthridine derivatives in the application set and their topoisomerase I inhibitory activity REC and classification results from the final SVM model.

Table S1. Chemical structure of 82 benzo[*c*]phenanthridine derivatives and their topoisomerase I inhibitory activity REC and classification results from final SVM model.

Classification: «+» presented stronger than topotecan; «–» presented weaker than topotecan

No	Chemical structure	Name	REC<br>Topo I mediated DNA cleavage (Experimental)	Topo I classified result (Predictive)
1	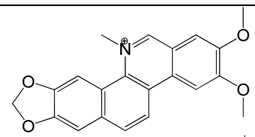	Nitidine	10	–
2	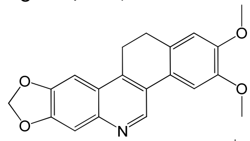	BMC_03_3795_10a	8	–
3	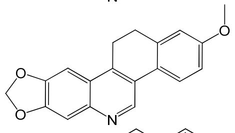	BMC_03_3795_10b	200	–
4	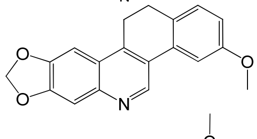	BMC_03_3795_10c	200	–
5	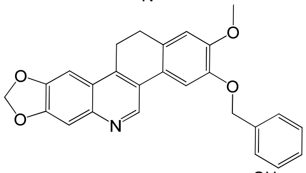	BMC_03_3795_10d	>1000	–
6	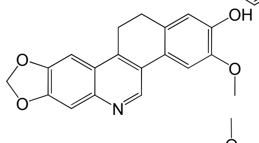	BMC_03_3795_10e	500	–
7	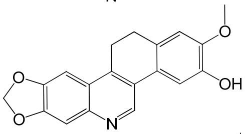	BMC_03_3795_10f	10	–
8	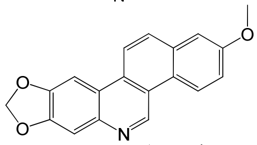	BMC_03_3795_11a	>1000	–
9	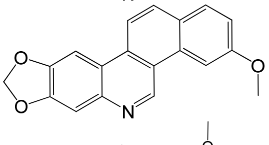	BMC_03_3795_11b	100	–
10	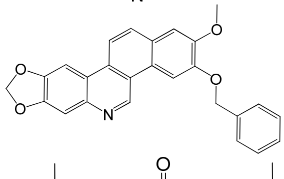	BMC_03_3795_12d	>1000	–
11	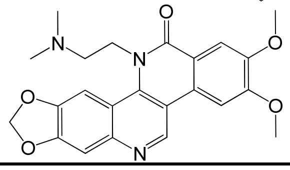	BMC_03_2061_03a	0.5	–

No	Chemical structure	Name	REC Topo I mediated DNA cleavage (Experimental)	Topo I classified result (Predictive)
12	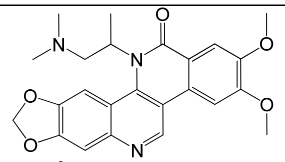	BMC_03_2061_03b	>1000	−
13	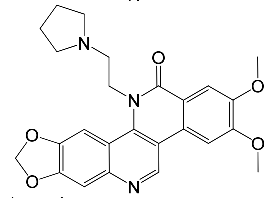	BMC_03_2061_03c	0.3	+
14	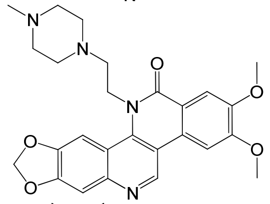	BMC_03_2061_03d	1000	−
15	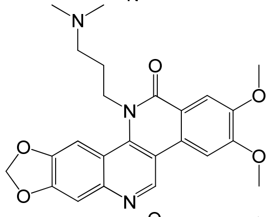	BMC_03_2061_03e	1	−
16	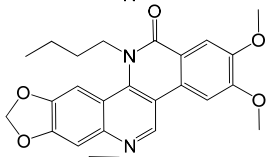	BMC_03_2061_03f	1000	−
17	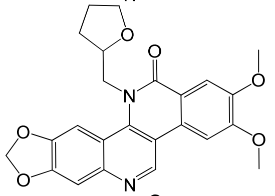	BMCL_02_3333_03c	10	−
18	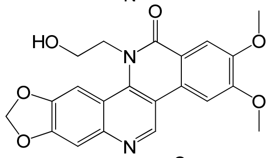	BMC_03_2061_03h	50	−
19	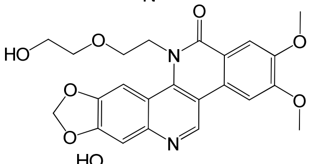	BMC_03_2061_03i	1	−
20	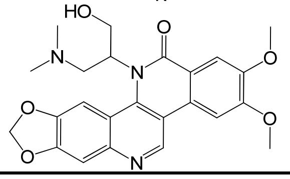	BMC_03_2061_03j	>1000	−

No	Chemical structure	Name	REC Topo I mediated DNA cleavage (Experimental)	Topo I classified result (Predictive)
21	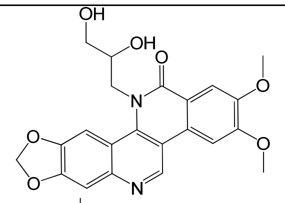	BMC_03_2061_03k	0.8−1.0	+
22	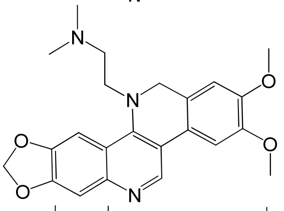	BMC_03_2061_04a	0.8	−
23	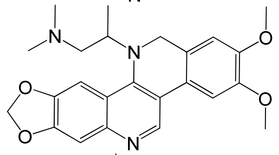	BMC_03_2061_04b	100	+
24	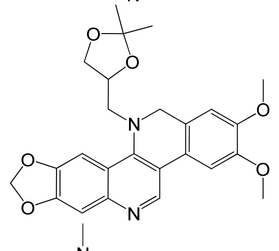	BMC_03_2061_09k	10	−
25	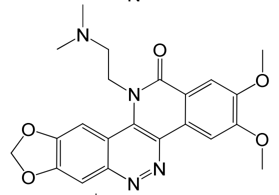	JMC_03_2254_02	0.3	−
26	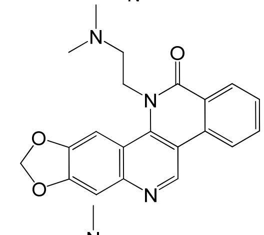	JMC_03_2254_03	6	−
27	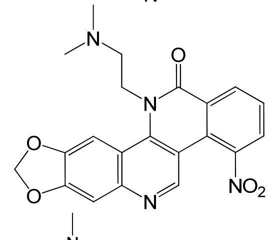	JMC_03_2254_05a	1000	+
28	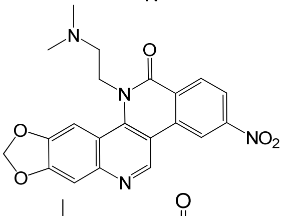	JMC_03_2254_05b	0.1	+
29	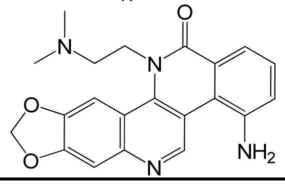	JMC_03_2254_06a	15	−

No	Chemical structure	Name	REC<br>Topo I mediated DNA cleavage (Experimental)	Topo I classified result (Predictive)
30	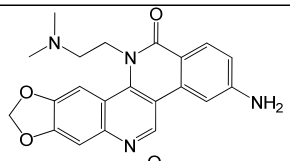	JMC_03_2254_06b	0.5	−
31	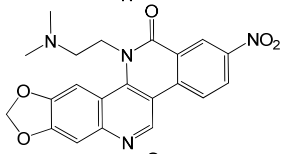	JMC_03_2254_05c	0.2	−
32	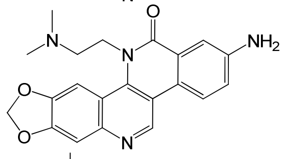	JMC_03_2254_06c	8.0	−
33	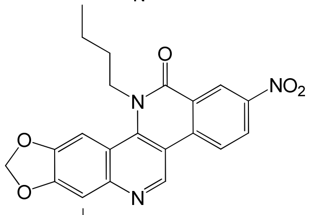	JMC_03_2254_16a	10	+
34	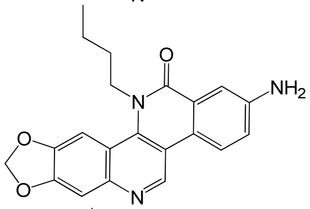	JMC_03_2254_17a	500	−
35	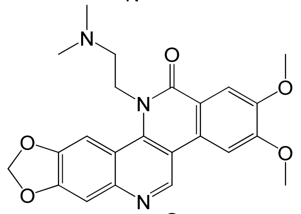	JMC_03_2254_02	0.5	+
36	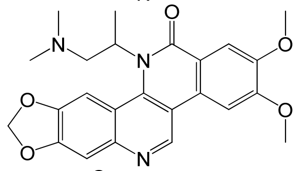	BMCL_02_3333_02	>1000	−
37	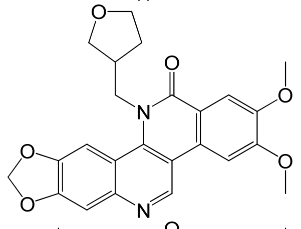	BMCL_02_3333_03	200	−
38	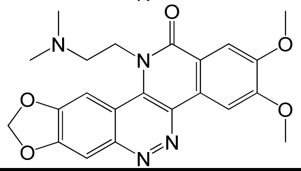	BMCL_02_3333_04a	0.3	+

No	Chemical structure	Name	REC Topo I mediated DNA cleavage (Experimental)	Topo I classified result (Predictive)
39	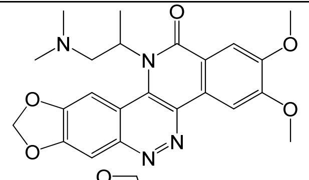	BMCL_02_3333_04b	1000	−
40	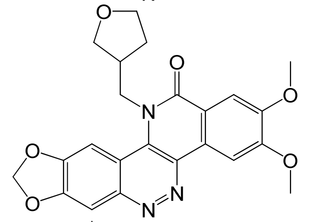	BMCL_02_3333_04c	30	−
41	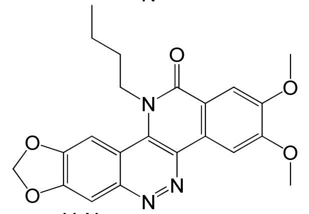	BMCL_02_3333_04d	1.0	−
42	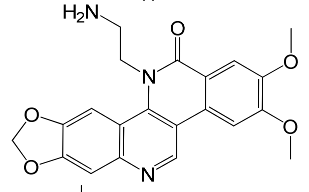	LDDD_04_198_01	0.03	−
43	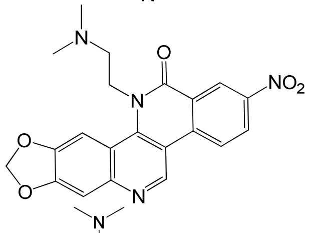	LDDD_04_198_02	2.0	−
44	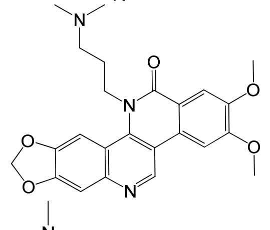	LDDD_04_198_03	2.0	−
45	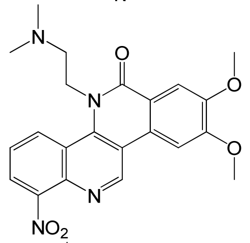	BMC_04_3731_03a	9	−
46	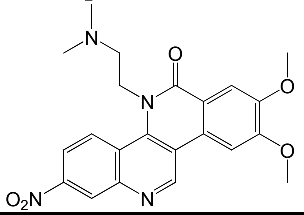	BMC_04_3731_03b	6	−

No	Chemical structure	Name	REC Topo I mediated DNA cleavage (Experimental)	Topo I classified result (Predictive)
47	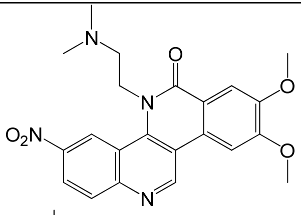	BMC_04_3731_03c	2	−
48	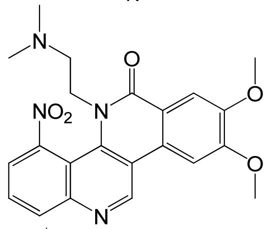	BMC_04_3731_03d	>300	−
49	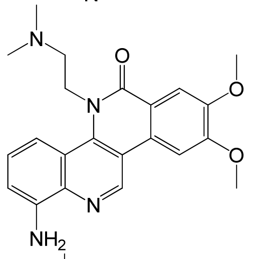	BMC_04_3731_04a	100	−
50	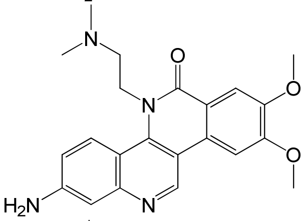	BMC_04_3731_04b	12	−
51	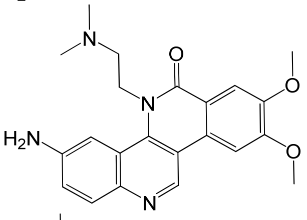	BMC_04_3731_04c	6	−
52	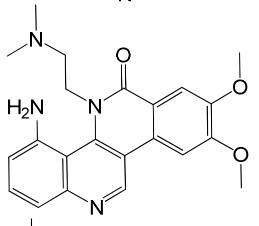	BMC_04_0795_04	>300	−
53	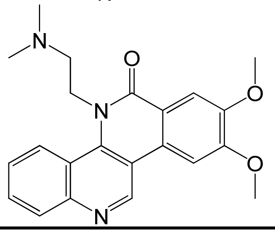	BMC_04_3731_05	10	−

No	Chemical structure	Name	REC Topo I mediated DNA cleavage (Experimental)	Topo I classified result (Predictive)
54	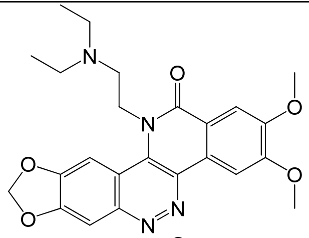	BMC_04_0795_01b	0.3	−
55	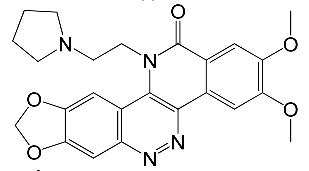	BMC_04_0795_01f	0.2	+
56	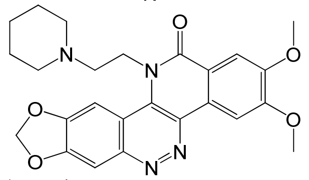	BMC_04_0795_01g	2.0	−
57	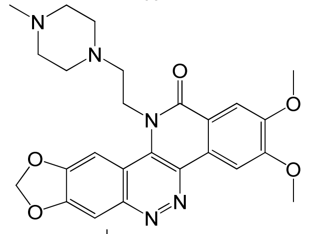	BMC_04_0795_01h	20	−
58	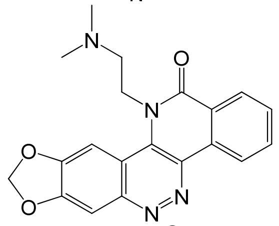	BMC_04_0795_03	10	−
59	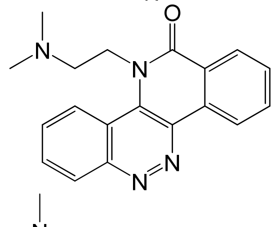	BMC_04_0795_02	>1000	−
60	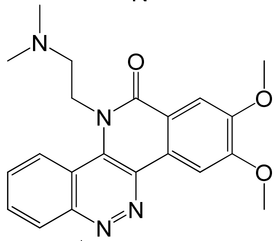	BMC_04_0795_04	0.8	+
61	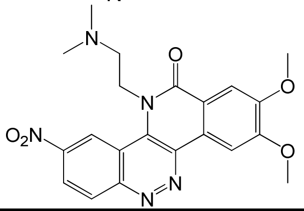	BMC_04_0795_05	5	−

No	Chemical structure	Name	REC Topo I mediated DNA cleavage (Experimental)	Topo I classified result (Predictive)
62	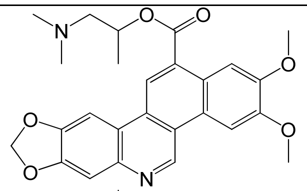	BMC_05_6782_07c	1.0	−
63	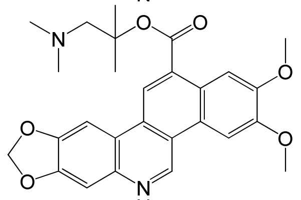	BMC_05_6782_07d	8	−
64	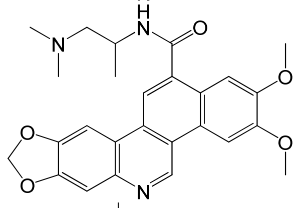	BMC_05_6782_08	0.6	+
65	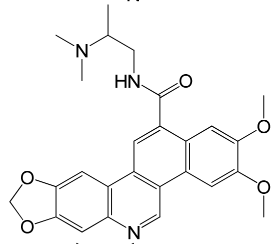	BMC_05_6782_09c	0.4	−
66	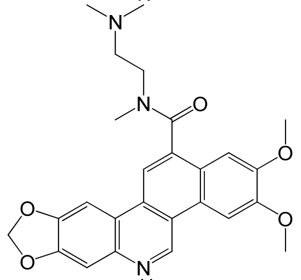	BMC_05_6782_09d	10	−
67	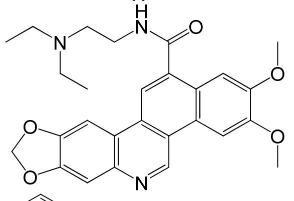	BMC_05_6782_09e	0.7	+
68	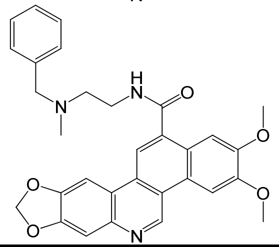	BMC_05_6782_09f	12	−

No	Chemical structure	Name	REC Topo I mediated DNA cleavage (Experimental)	Topo I classified result (Predictive)
69	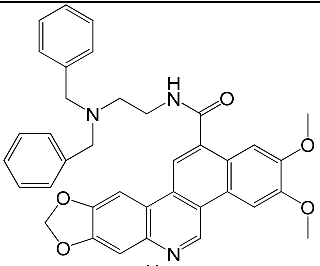	BMC_05_6782_09g	60	−
70	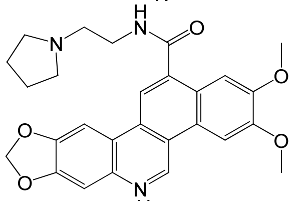	BMC_05_6782_09h	0.6	+
71	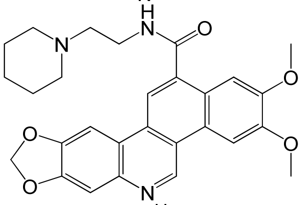	BMC_05_6782_09i	1.5	−
72	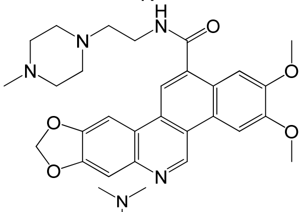	BMC_05_6782_09j	0.3	+
73	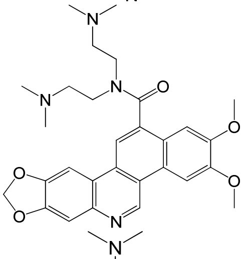	BMC_05_6782_10	3	+
74	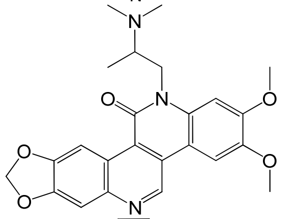	BMC_06_3131_10c	1.2	−
75	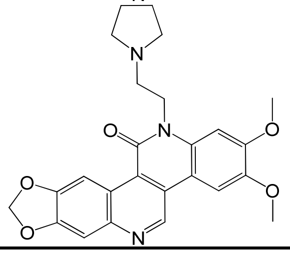	BMC_06_3131_10d	0.07	+

**No**	**Chemical structure**	**Name**	**REC Topo I mediated DNA cleavage (Experimental)**	**Topo I classified result (Predictive)**
76	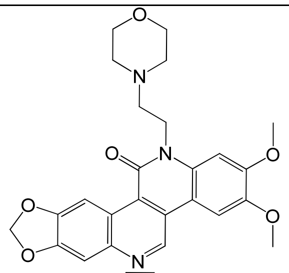	BMC_06_3131_10g	9	−
77	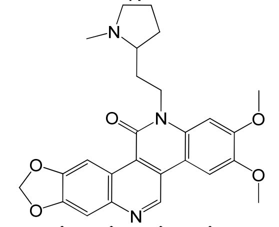	BMC_06_3131_10i	0.45	+
78	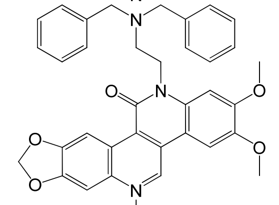	BMC_06_3131_10j	>100	−
79	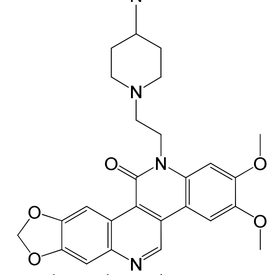	BMC_06_3131_10f	3	−
80	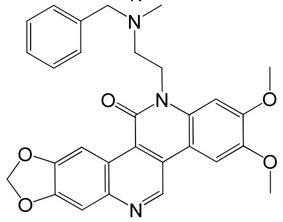	BMC_06_3131_10k	13	−
81	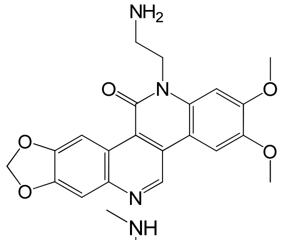	BMC_06_3131_10l	0.35	+
82	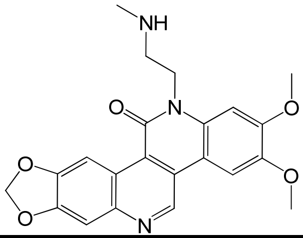	BMC_06_3131_10m	0.15	+

Table S2. Chemical structure of 10 benzo[*c*]phenanthridine derivatives in application set and their topoisomerase I inhibitory activity REC and classification results from final SVM model.

Classification: «+» presented stronger than topotecan; «–» presented weaker than topotecan

No	Chemical structure	Name	REC<br>Topo I mediated<br>DNA cleavage<br>(Experimental)	Topo I<br>classified<br>result<br>(Predictive)
A1-<br>A2	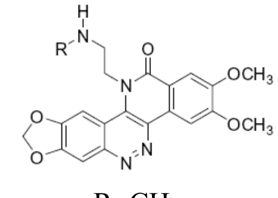<br>R=CH_3<br>R=$CH(CH_3)_2$	BMC_08_7824_7a<br>BMC_08_7824_7b	0.03<br>0.08	+<br>+
A3	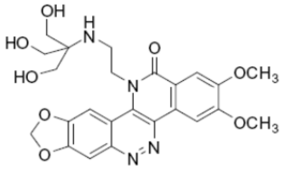	BMC_08_7824_9	0.2	+
A4	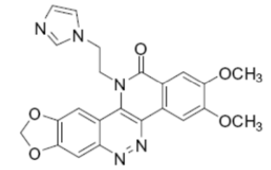	BMC_08_7824_11	0.1	+
A5	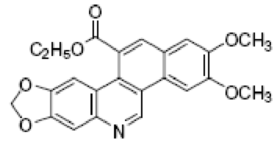	BMC_08_8598_9	>10	–
A6	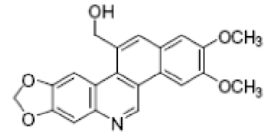	BMC_08_8598_10	0.2	–
A7	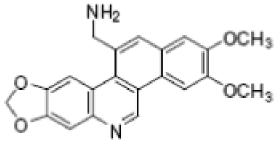	BMC_08_8598_12	0.2	–
A8	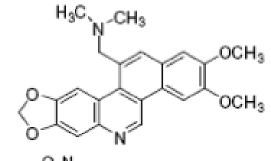	BMC_08_8598_13	0.2	+
A9	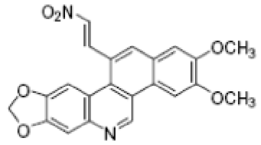	BMC_08_8598_14	>10	–
A10	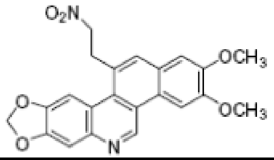	BMC_08_8598_15	>10	–
